# Genome Sequence of the Attenuated Equid Alphaherpesvirus 1 Strain AK-2011, Isolated in Kazakhstan

**DOI:** 10.1128/mra.01328-22

**Published:** 2023-02-14

**Authors:** Kobey Karamendin, Abdikalyk Abishov, Aidyn Kydyrmanov, Moldir Akhmetzhanova

**Affiliations:** a Laboratory of Viral Ecology, Scientific Production Center of Microbiology and Virology, LLP, Almaty, Kazakhstan; b Virology Laboratory, SPE DiaVak-ABN, LLP, Almaty, Kazakhstan; c Virology Laboratory, Antigen, LLP, Almaty, Kazakhstan; d Kazakh National Agrarian Research University, Almaty, Kazakhstan; DOE Joint Genome Institute

## Abstract

In 2011, there was an outbreak of a disease with mass abortions among horses in southeastern Kazakhstan. The AK-2011 strain was isolated from an aborted fetus and subsequently identified as equid alphaherpesvirus 1. Here, we describe the nearly complete genome sequence of the AK-2011 strain, attenuated for vaccine development.

## ANNOUNCEMENT

Equid alphaherpesvirus 1 (EHV-1 [formerly equine herpesvirus 1]) belongs to the genus *Varicellovirus*, subfamily *Alphaherpesvirinae*. It is associated with upper respiratory tract disease, abortion, and, in some cases, neurological disease (1). The EHV-1 strain AK-2011 was isolated from multiple tissue samples from an aborted fetus on primary lamb kidney cells. Briefly, the cell monolayer was inoculated with the filtered organ extracts and incubated in a 5% CO_2_ environment at 37°C for 1 h. The inoculum was removed, and the monolayer was rinsed with maintenance medium. After addition of supplemented maintenance medium, the cells were incubated at 37°C for 2 weeks until viral cytopathic effect (CPE) was observed. During the incubation period, the maintenance medium was replaced every 3 days. The virus was then attenuated by 10 serial passages on a calf trachea cell line (2).

The cell culture supernatant was concentrated using Amicon Ultra-4 filter units (Merck Millipore, Ireland). Viral DNA was extracted using the QIAamp viral RNA minikit (Qiagen, Germany) according to the manufacturer’s protocol. The DNA obtained was fragmented to 400 to 600 bp using a DNA fragmentation kit (New England Biolabs, USA). The library was constructed using the NEBNext Ultra DNA library preparation kit (New England Biolabs) following the manufacturer’s instructions. Library quality and quantity were assessed with a 2100 Bioanalyzer (Agilent, Germany) and a Qubit 2.0 fluorometer (Invitrogen, USA), respectively. Paired-end sequencing was performed on a MiSeq instrument using the version 3 reagent kit (Illumina). The quality of the reads obtained was assessed using FastQC (3). Default parameters were used for all software unless otherwise specified. In total, 4,243,858 raw sequencing reads, with a mean length of 221 nucleotides, were obtained. The reads obtained were trimmed at the 3′ and 5′ ends with an error probability limit of 0.05 and *de novo* assembled using the SPAdes assembler (4) in the Geneious Prime software package (Biomatters, New Zealand) (5). Finally, the raw sequencing reads and *de novo* assembled contigs were mapped on the 99.89% genetically closest EHV-1 genome sequence (determined by a nucleotide BLASTn search) of the RacL 11 strain downloaded from GenBank (accession number MF975656) using Geneious Prime (medium sensitivity, four iterations) to produce a consensus sequence. All of the assemblies were visually inspected, and misassemblies were manually corrected.

Typically, EHV-1 has a linear double-stranded DNA (dsDNA) genome of about 150 kbp in size, with a GC content of about 56.7% (6). The genome contains 80 open reading frames (ORFs) with 76 different genes; 4 duplicated ORFs are located in the terminal repeat sequence (7). The final genome sequence obtained for the AK-2011 strain contained 145,607 nucleotides, with a mean coverage of 3,159-fold and a GC content of 56.5%. Genome annotation resulted in a total of 75 predicted protein-encoding genes. The IR6 gene (ORF67; virulence factor) was deleted, compared to the genetically closest RacL11 genome. Mutations in the IR4 (ORF65; host range determinant, cotransactivator), US1 (ORF68; virion protein), and US5 (ORF72; glycoprotein D) genes were detected in the AK-2011 genome. Supposedly, some of these deletions and mutations as a result of serial passages in cell lines lead to the loss of virulence *in vivo*, as described previously (8, 9).

### Data availability.

The nearly complete genome sequence of the AK-2011 strain is available in GenBank under accession number OP271695. Raw sequence reads were deposited under BioProject accession number PRJNA873220, and the SRA accession number of the library is SRR21711097.
